# Surgical Navigation and CAD-CAM-Designed PEEK Prosthesis for the Surgical Treatment of Facial Intraosseous Vascular Anomalies

**DOI:** 10.3390/jcm13164602

**Published:** 2024-08-06

**Authors:** Alicia Dean, Orlando Estévez, Concepción Centella, Alba Sanjuan-Sanjuan, Marina E. Sánchez-Frías, Francisco J. Alamillos

**Affiliations:** 1 Maxillofacial Surgery Department, Reina Sofía University Hospital, Maimonides Institute for Biomedical Research of Córdoba (IMIBIC), 14004 Cordoba, Spain; orlando_estevez@hotmail.com (O.E.); centellaguti@hotmail.com (C.C.); drfalami@gmail.com (F.J.A.); 2 Maxillofacial Surgery Department, Charleston Area Medical Center, Charleston, WV 25301, USA; albasanjuan@hotmail.com; 3 Pathology Department, Reina Sofía University Hospital, Maimonides Institute for Biomedical Research of Córdoba (IMIBIC), 14004 Cordoba, Spain; marinasanchezfrias@gmail.com

**Keywords:** surgical navigation, virtual surgery, CAD-CAM design, computed-assisted surgery, virtual planning, computer-assisted navigated piezoelectric surgery, 3D planning

## Abstract

**Background**: Intraosseous vascular anomalies in the facial skeleton present significant diagnostic and therapeutic challenges due to complex anatomy. These anomalies represent about 0.5–1% of bony neoplastic and tumor-like lesions, usually presenting as a firm, painless mass. Most described intraosseous vascular malformations are venous malformations (VMs) and, more rarely, arteriovenous malformations. **Objectives**: The objectives of this work are to show our experience, protocol and the applications of computer planning, virtual surgery, CAD-CAM design, surgical navigation, and computer-assisted navigated piezoelectric surgery in the treatment of facial intraosseous vascular anomalies and to evaluate the advantages and disadvantages. **Methods**: Three females and one male with periorbital intraosseous vascular anomalies were treated using en-block resection and immediate reconstruction with a custom-made PEEK prosthesis. One lesion was in the supraorbital rim and orbital roof, one in the frontal bone and orbital roof, and two in the zygomatic region. We accomplished the resection and reconstruction of the lesion using virtual planning, CAD-CAM design, surgical navigation and piezoelectric device navigation. **Results**: There were no complications related to the surgery assisted with navigation. With an accuracy of less than 1 mm, the procedure may be carried out in accordance with the surgical plan. The surgeon’s degree of uncertainty during deep osteotomies and in locations with low visibility was decreased by the use of the navigated piezoelectric device. **Conclusions**: Resection and reconstruction of facial intraosseous vascular anomalies benefit from this new surgical strategy using CAD-CAM technologies, computer-assisted navigated piezoelectric surgery, and surgical navigation.

## 1. Introduction

Intraosseous vascular anomalies of the facial skeleton present a significant diagnostic and therapeutic challenge due to its complex anatomy [1].

According to Mulliken and Glowacki, there are two types of vascular anomalies: vascular tumors and vascular malformations [2]. The 2018 International Society for the Study of Vascular Anomalies (ISSVA) classification does not accept the diagnosis of intraosseous hemangioma, distinguishing between proliferative lesions (tumors) and non-proliferative lesions (malformations) [3,4,5].

Most described intraosseous vascular malformations are venous malformations (VMs) and more rarely arteriovenous malformations (AVMs). Many have been erroneously labeled as “hemangiomas” [1,5].

These anomalies represent about 0.5–1% of all bony neoplastic and tumor-like lesions and mostly affect the vertebral column and calvarium [6,7]. Maxillofacial involvement is uncommon, with the maxilla and mandible being the most frequently affected sites [6,7,8]. Zygomatic bone, nasal, and frontal bone involvement has also been described [9,10,11,12].

They typically occur in the fourth to fifth decade, affecting both genders almost equally [5,7], although some studies note a female predominance [6,9]. Trauma is a commonly suggested cause [13,14,15], although congenital factors are also considered [16,17,18]. VMs present as a firm, painless lump or mass [1,7,19,20] and can cause swelling or pain in the maxilla and the mandible, or neurological symptoms when in the sphenoid bone [21] or cranial base [22,23]. Rarely, they can cause tooth displacement [7]. Lesions of periorbital bones usually produce asymptomatic contour defects with esthetic compromise [6,8,10,24,25,26]. When symptomatic, pain (49%) and subsequent by ocular features (14.2%) related to the mass effect (dystopia, exorbitism, ptosis and extraocular muscle movement impairment) are the most common symptoms [9,25,27,28,29,30,31,32,33]. 

VMs show a tendency to grow with time and can worsen due to trauma, infection, puberty or pregnancy [7,11,34]. Traumatic injury may predispose to periods of accelerated growth; however, this association remains controversial [6].

There may be extensive bleeding during biopsy or surgery; so, it is imperative to suspect the vascular nature of the lesion [9]. 

On plain radiography, VMs are slightly radiopaque and usually well circumscribed [35]. On CT, the lesions may appear with honeycomb, soap bubble, or sunburst patterns [6,15,18,28,34,35,36,37]. On MRI, VMs appear hypo- to isointense on T1 images and hyperintense on T2 images. In orthopantomography and plain radiography, AVMs appear as radiolucent lesions, and CT scans show them as expansive lytic defects that are vividly enhanced on contrast administration [35,38]. On MRI, an AVM appears as a tangle of vessels with a typical flow–void phenomenon, seen on both T1- and T2-weighted imaging [1,29,34,38]. CT is the standard radiological study. However, several authors prefer MRI to CT [13,18,39].

Histopathology differentiates VMs, characterized by abnormal thin-walled veins, from AVMs, with arteriovenous shunts [6].

Immunohistochemical staining also aids in distinguishing these lesions: VMs are positive for CD31 and CD34, AVMs for smooth muscle actin, and hemangiomas for GLUT-1 [6].

Treatment depends on symptoms, location, and extent.

Complete surgical resection is its treatment of choice [7,8,19,24,40], reducing recurrence [40], and bleeding risks [9,26]. Preoperative arteriography and embolization may be beneficial for high-flow lesions. 

Advanced technologies like virtual planning, surgical navigation, and computer-assisted navigated piezoelectric surgery (CANPS) enhance precision and outcomes and minimize complications and surgical approaches in the surgical treatment of facial intraosseous vascular anomalies [41,42,43,44,45,46,47]. 

Computer-assisted navigated piezoelectric surgery (CANPS) applies computer planning and surgical navigation to a piezoelectric device so that it becomes navigable [48,49].

The main objective of this work is to show our experience, our protocol, and the applications of computer planning, virtual surgery, CAD-CAM design, surgical navigation and computer-assisted navigated piezoelectric surgery in the treatment of facial intraosseous vascular anomalies and to evaluate the advantages and disadvantages of this innovative surgical approach.

## 2. Materials and Methods

### 2.1. Patients

The study design is based on a descriptive study (case series) of 4 patients with intraosseous vascular anomalies treated in the Department of Craniomaxillofacial Surgery at a tertiary referral hospital between June 2017 and May 2019. All patients provided informed written consent to accomplish the surgery and posteriorly also to participate in the study.

The inclusion criteria were as follows: (1) presence of a craniomaxillofacial intraosseous vascular anomaly; (2) resection and reconstruction of the tumor using virtual planning, CAD-CAM design and surgical navigation; and (3) minimum follow-up of 1 year. The exclusion criteria were as follows: (1) resection of intraosseous vascular anomaly without virtual planning, CAD-CAM design and surgical navigation; and (2) incomplete records.

### 2.2. Data Collection

Data collection was carried out based on a protocol established before the study began and reviewed at the end of it. Clinical variables related to the patients were recorded. The variables and data collection form are attached in Table 1.

### 2.3. Procedure

En-block resection and immediate custom-made PEEK prosthesis reconstruction were planned for all four patients.

#### 2.3.1. Virtual Surgical Plan

DICOM (Digital Imaging and Communication in Medicine) files from CT helical scan with 0.8 mm thin slices data were imported into the planning BrainLab software, iPlan^®^ 3.0.6 and Elements^®^ 4.0 (Munich, Germany). A virtual surgery with a treatment plan was carried out. The vascular malformation shape was outlined, and an appropriate 1mm surgical margin was automatically created using the tool “margin”. The object “lesion resection” was created and the virtual bone defect was evaluated after the virtual resection was performed on the computer. Essential anatomical structures to be protected from injury during surgery were also delimited and marked.

#### 2.3.2. CAD-CAM Design of the PEEK Prosthesis

The object “lesion resection” was converted into .STL files that were sent to Materialise^®^ (Materialise, Leuven, Belgium; https://www.materialise.com/en, accessed on 31 July 2024), which built a custom-made PEEK prosthesis. The prosthesis was manufactured by mirroring the healthy side and following surgical resection margins. The surgical plan, resection guide, and prosthesis were converted into .STL files. The files were sent back to us and imported into the iPlan^®^ 3.05 software or Elements^®^ planning software of the BrainLab^®^ navigation system. The .STL files of the plan were superimposed onto patient-specific CT scan data to check the accuracy of the resection and PEEK reconstruction plan. 

#### 2.3.3. Surgical Navigation

Surgical navigation with the BrainLab^®^ system was used to guide the resection. There are three moments of surgical navigation: anatomical navigation (1st navigation), working navigation (2nd navigation), and checking navigation (3rd navigation). Anatomical navigation checks anatomical structures, working navigation helps with surgical plans, and checking navigation or simulation-guided navigation verifies reconstruction [49,50]. CANPS is a type of working navigation [49,50].

A skull post with a dynamic reference frame was fixed to the patient’s skull. Patient registration was performed using the surface laser or unequivocal bone points.

Registration and calibration of the cutting tip of the piezoelectric device were carried out. The piezoelectric handpiece was registered by anchoring the three reflecting spheres tracking tool to the handpiece of a Vercellotti-type piezoelectric device and linking it to the navigator with a calibration matrix. The cutting guides were set in position and anchored with screws onto the healthy bone. The osteotomy of the lesion was performed with a piezoelectric device following the custom-made surgical guides over the bone surface and then in depth in the orbital roof, orbital floor and orbital walls with the aid of the CANPS. We accomplished 1. Indirect, and 2. Direct or “live” surgical navigation. We performed live piezoelectric navigation with real-time results on the workstation screen. The precision of the piezoelectric device’s cutting tip was monitored before and throughout the surgery. Accuracy was ensured by positioning the calibrated cutting tip on specific anatomical landmarks. Surgery was carried out with a precision of 1 mm. If deviations exceeded this limit, the device was re-registered and recalibrated. After the resection, the navigation again helped us check the planned resection margins.

The PEEK prostheses were placed in position for reconstruction. Patient-specific implants did not require additional adaptation or remodeling because navigation-assisted resection ensured precise excision of the deep margins according to the preoperative plan. In Case 1, a groove was created for the passage of the pericranial flap to isolate the frontal sinus and nasal cavities from the skull base and prosthesis. This groove, approximately 3 mm thick, prevents compression over the pericranial flap when placing the PEEK prosthesis. The customized PEEK prostheses were fixed in all cases with titanium mini-plates. Osteosynthesis was performed using conventional osteosynthesis systems (Matrix Midface^®^) with low-profile mini-plates to prevent them from being palpable under the skin. Two holes and screws at least on each side of the osteosynthesis points were used. Navigation was performed again to recheck the PEEK prosthesis’s accurate position, known as the third navigation or “Simulation-Guided Navigation” (SGN). 

Accuracy was postoperatively verified by superimposing the postoperative CT scan onto the preoperative CT scan, which contained the surgical plan. Measurements were then taken in the axial, sagittal, and coronal planes.

This study was conducted in accordance with the tenets of the WMA Declaration of Helsinki in the context of Ethical Principles for Medical Research Involving Human Subjects. It was approved by our institution’s local institutional review board (Act. number 301, ref. 4626; 03-2020).

## 3. Results

The study sample comprised three females and one male, with an average age of 47.5 years old. One lesion was in the supraorbital rim and orbital roof, one in the frontal bone and orbital roof and two in the zygomatic region (Table 1). CANPS was successfully performed.

There were no complications related to navigated surgery. The surgery could be performed according to the surgical plan with a precision of 1 mm.

The use of the navigated piezoelectric device reduced the surgeon’s uncertainty during the osteotomies in depth and in poorly visible areas. Three experienced surgeons, two maxillofacial surgeons and one plastic surgeon with experience in facial reconstruction, independently assessed the esthetic result as excellent in all four patients. The evaluation was performed using a five-choice graded scale: poor, fair, satisfactory, very satisfactory and excellent. Figure 1, Figure 2, Figure 3, Figure 4, Figure 5, Figure 6, Figure 7, Figure 8 and Figure 9 represent Case 2. Figure 10, Figure 11, Figure 12, Figure 13, Figure 14, Figure 15, Figure 16, Figure 17 and Figure 18 illustrate Case 3.

## 4. Discussion

The use of virtual surgical planning, CAD-CAM design of prostheses and customized surgical guides has been applied by a few authors for the surgical management of vascular anomalies of the facial bones [9,41,51,52]. To the best of our knowledge, no paper has combined surgical navigation with these technologies. Moreover, it is also the first time that both indirect surgical navigation with a pointer and direct surgical navigation with the piezoelectric device have been performed to resect vascular anomalies of facial bones.

Intraosseous vascular anomalies of the facial skeleton represent a diagnostic and therapeutic challenge for craniofacial surgeons. Computer-assisted technology for virtual planning, CAD-CAM designed PEEK prosthesis, surgical navigation and piezoelectric resection represent a new trend in the multidisciplinary treatment of these complex anomalies. This technology improves cutting precision and intraoperative safety, helping to minimize esthetically impairing scars and surgical morbidity, achieving exceptional outcomes in this anatomical region.

Careful clinical and radiographic evaluation is essential to avoid misdiagnosing hemangiomas, venous malformations, and arteriovenous malformations. Histopathological and immunohistochemical analyses may be required in some cases. 

We, as many authors, recommend immediate reconstruction after surgical resection. Common reconstructive options include autogenous calvarial graft [27,53,54], pre-bent titanium mesh on a standard or a patient stereolithographic model [19,55], and customized PEEK (Polyetheretheretherketone), titanium, methyl-methacrylate prostheses or polycaprolactone/beta tricalcium phosphate scaffold [9,12,51,52,56,57,58,59,60,61,62]. Customized prostheses allow for a reliable reconstruction with excellent esthetic results, avoid morbidity in the donor site, and reduce surgical time.

When comparing reconstruction materials, PEEK has similar strength and weight to human bone, offers high biocompatibility and durability, is radiolucent and has low rates of infection and allergic reactions, but can be expensive and has a higher infection rate compared to titanium. Titanium prostheses are strong, biocompatible and have superior osseointegration potential, but are radiopaque, expensive and difficult to modify during surgery. Both PEEK and titanium can be sterilized and customized. PEEK mimics bone elasticity and density better, is adjustable during surgery, and can increase in thickness to restore volume, but requires titanium screws for fixation due to its poor osseointegration. Autologous bone grafts integrate well biologically with minimal rejection, but may cause donor site morbidity and are in limited availability. PMMA is a cost-effective and easy to shape bone cement, but presents a higher risk of infection, especially for long-term use [60,61,62].

Several authors have reported that the use of virtual surgery and CAD-CAM design of prostheses and customized surgical guides improves the accuracy of the reconstruction and its esthetic results, reduces complications of ablation and reconstruction, and decreases surgical time and postoperative hospital stay [12,51,57]. In our cases, we could establish that there was less than 1 mm of difference between the planned resection and reconstruction with the postoperative CTs. The esthetic results were excellent in all cases according to the surgeon’s and patients’ appreciation. Shorter surgeries minimize the risk of infection and other intraoperative complications, while shorter hospital stays benefit patient recovery. From our point of view, the integration of virtual surgery, CAD-CAM design and surgical navigation technology represents a significant advancement in reconstructive surgery [42,43,44,45,46,47,59].

According to the two types of navigation methods, “indirect” or “sequential” navigation uses a probe intermittently. In contrast, “direct”, live, continuous, or real-time navigation” registers the operating instrument as a probe, allowing for continuous navigation during surgery. CANPS is a “direct” navigation [49,50]. CANPS can be applied to guide osteotomies in depth and in areas where it is impossible to place surgical guides because of their limited accessibility. CANPS can be used to control these hidden osteotomies during the resection of facial intraosseous vascular malformations [48,49]. Navigation can be once more used to verify the precise placement of the PEEK prosthesis, a process referred to as the third navigation or “Simulation-Guided Navigation” (SGN).

The advantages of computer planning and surgical navigation in treating intraosseous vascular malformations include accurate preoperative diagnosis, virtual surgery simulation, improved reconstruction accuracy, increased surgical safety, “direct navigation” or real-time guidance during surgery, reducing the risk of injury to anatomical structures, and operative time. The use of the navigation systems saves overall surgical time by decreasing uncertainty and increasing the surgeon’s confidence and precision with the resection and the reconstruction [41,43,45,46,47,59].

Possible complications related to virtual planning and surgical navigation are as follows: errors in planning and establishing appropriate surgical resection margins, loss of accuracy during surgical navigation, loosening of the screw that fixes the skull post with the dynamic reference frame to the skull, and inadvertent injuries with the navigation instruments.

We describe a novel surgical strategy for facial intraosseous vascular anomalies, using minimally invasive resection with piezosurgery and surgical navigation. It provides precise and confident resection and reconstruction but has an initial cost and learning curve drawbacks. Navigation saves time by decreasing uncertainty but requires preoperative planning and takes up space in the operating room.

## 5. Conclusions

Resection of facial intraosseous vascular anomalies can benefit from using CAD-CAM technologies, Computer-assisted navigated piezoelectric surgery and surgical navigation. CAD-CAM allows for the manufacture of PEEK prostheses that can be immediately adapted to the defect. Surgical navigation allows for the performance of osteotomies according to the planning, maximizing surgical precision and safety.

## Figures and Tables

**Figure 1:**
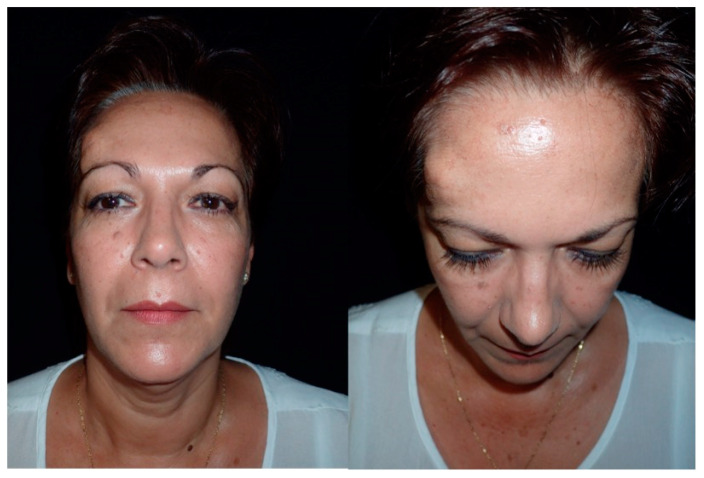
These images show the preoperative external appearance of the face of Patient 2. The protrusion can be seen in the right frontal region due to an intraosseous venous malformation.

**Figure 2:**
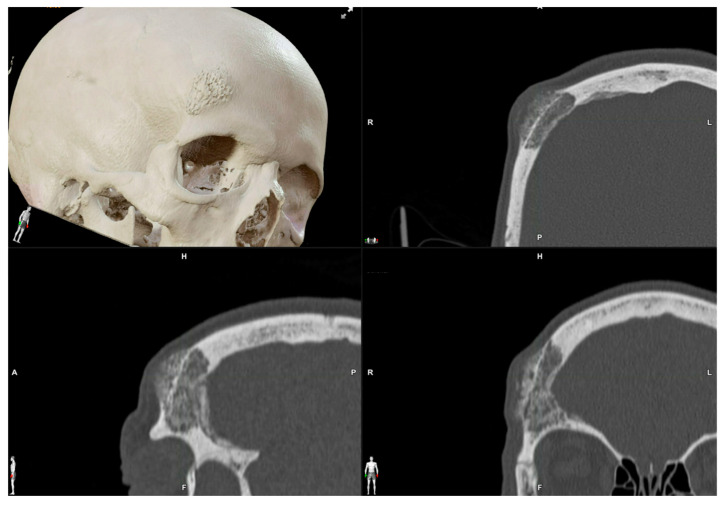
Preoperative CT scan. Three-dimensional, axial, sagittal and coronal views can be appreciated. There is a mixed radiolucent lesion and an expansive lytic defect affecting the frontal bone above the right supraorbital rim and roof of the right orbit. A: anterior, P: posterior, H: head, F: foot, R: right, L: left.

**Figure 3:**
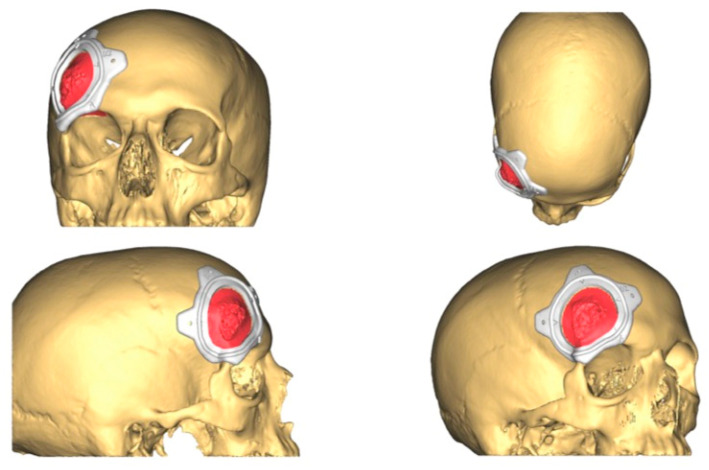
The resection plan (in red in the figure) and the surgical cutting guide that will conduct the resection on the surface of the frontal bone as planned.

**Figure 4:**
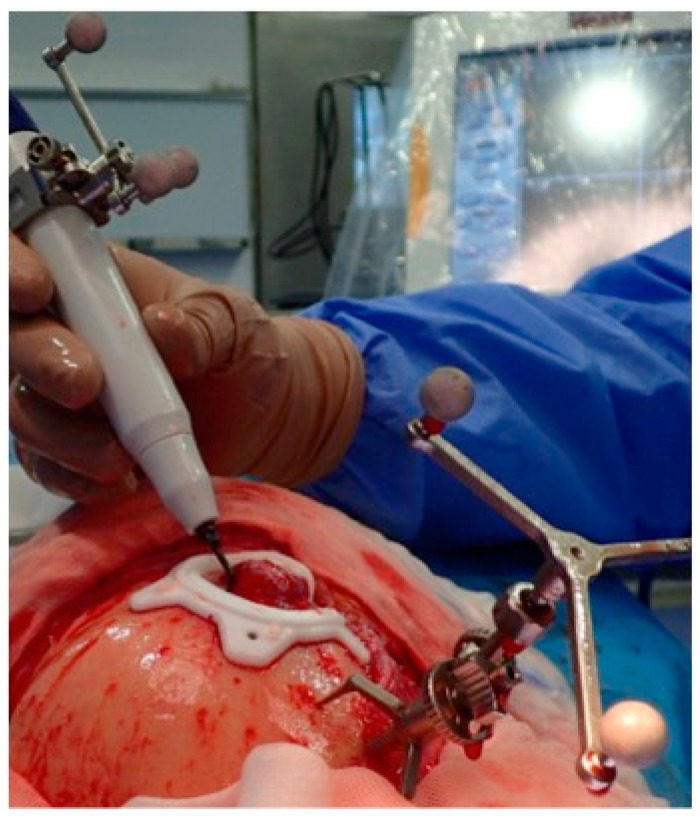
Navigated piezoelectric device. Direct or “live” navigation of the frontal bone. The skull post was anchored with a self-drilling screw.

**Figure 5:**
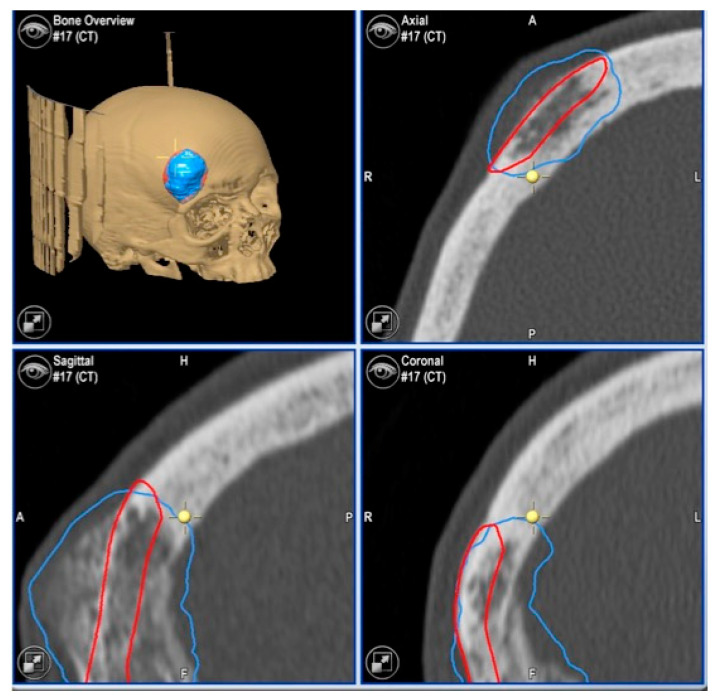
Images of direct navigation with the piezoelectric device (yellow dot with small cross) are shown on the navigation screen. The progression and depth of the guided osteotomy can be appreciated and evaluated in real time (“live navigation”). A: anterior, P: posterior, H: head, F: foot, R: right, L: left. In blue, the virtual resection; in red, the virtual reconstruction with the PEEK prosthesis.

**Figure 6:**
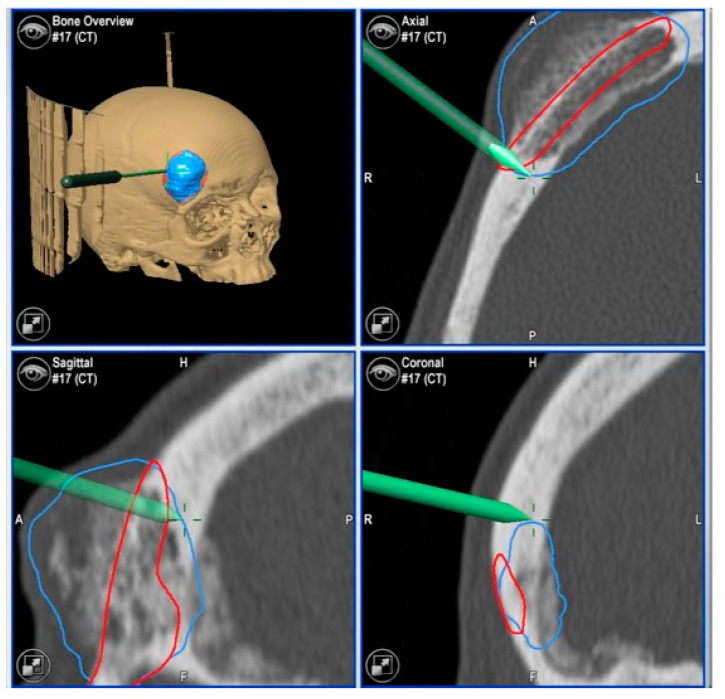
With the navigation pointer, the osteotomy (indirect navigation) we perform (green line on the navigation screen) can also be checked. A: anterior, P: posterior, H: head, F: foot, R: right, L: left. In blue, the virtual resection; in red, the virtual reconstruction with the PEEK prosthesis.

**Figure 7:**
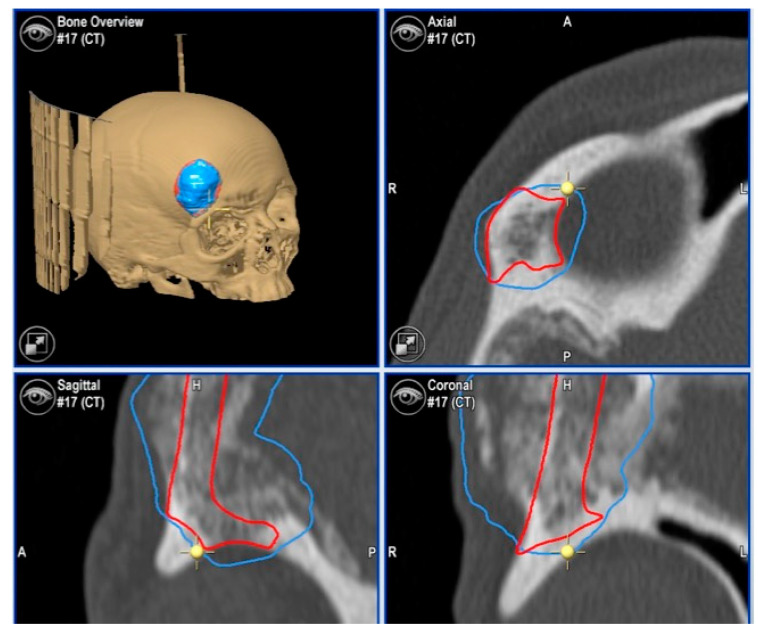
Images of direct navigation of the right orbital roof with the navigated piezoelectric device (yellow dot with small cross) are shown on the navigation screen. The progression and depth of the guided osteotomy can be appreciated and evaluated in real time (“live navigation”). A: anterior, P: posterior, H: head, F: foot, R: right, L: left. In blue, the virtual resection; in red, the virtual reconstruction with the PEEK prosthesis.

**Figure 8:**
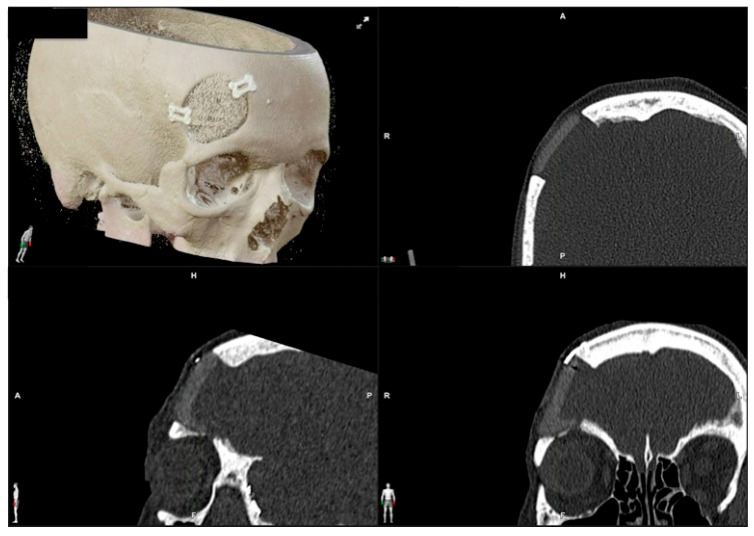
Postoperative CT scan. Three-dimensional, axial, sagittal and coronal views can be appreciated.

**Figure 9:**
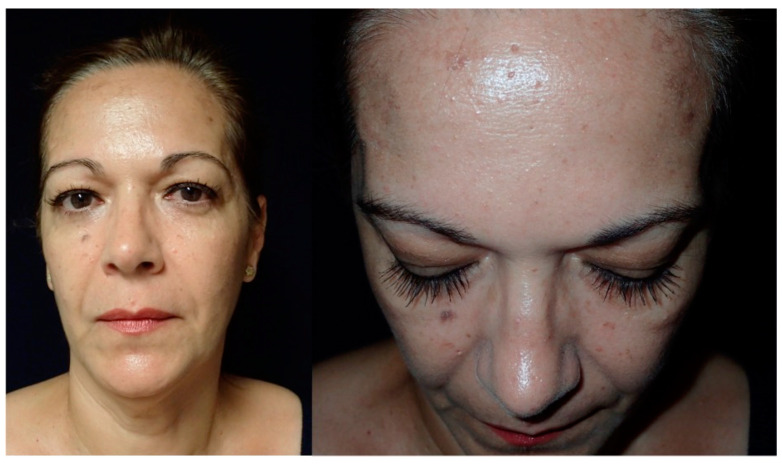
These images show the postoperative facial appearance with reestablishment of the frontal contour.

**Figure 10:**
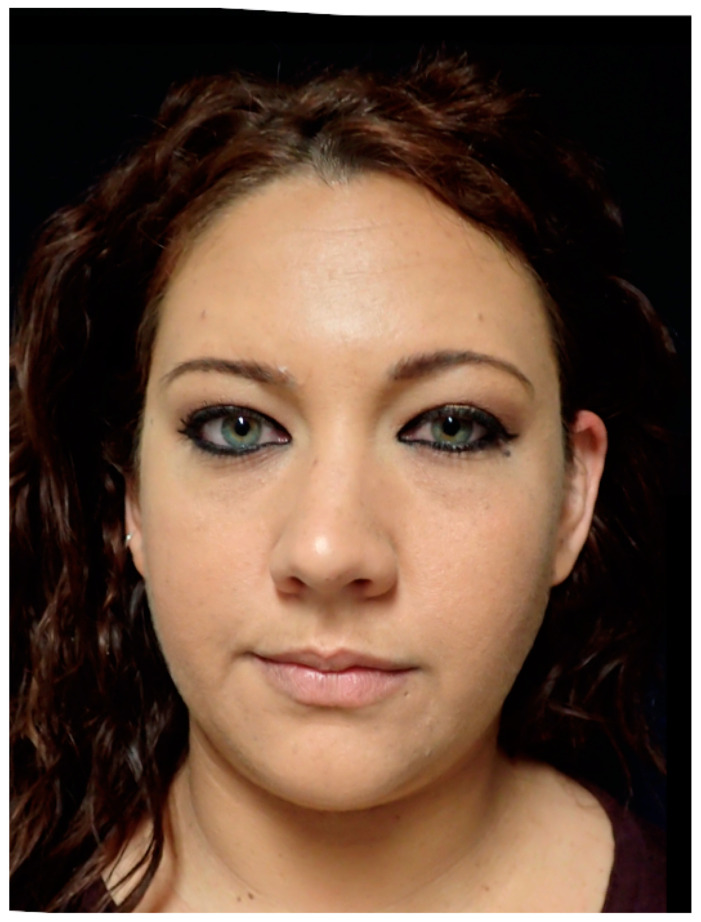
This image shows the preoperative external appearance of the face of Patient 3. There is a slight elevation of the left eyeball and a protrusion of the left cheek.

**Figure 11:**
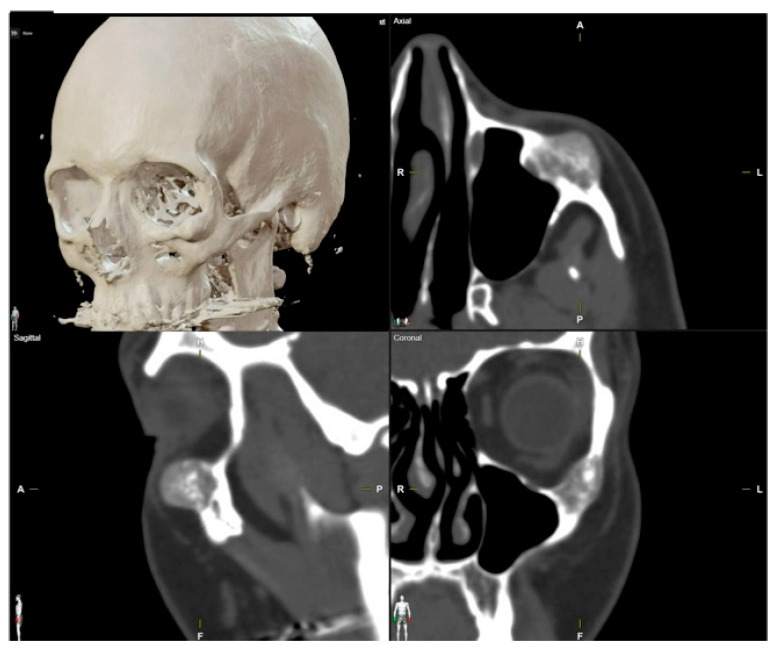
Preoperative CT scan. Three-dimensional, axial, sagittal and coronal views can be appreciated. There are a mixed radiolucent lesion and an expansive lytic defect affecting the left infraorbital rim, zygoma, external zone of the orbital floor and inferior part of the external wall.

**Figure 12:**
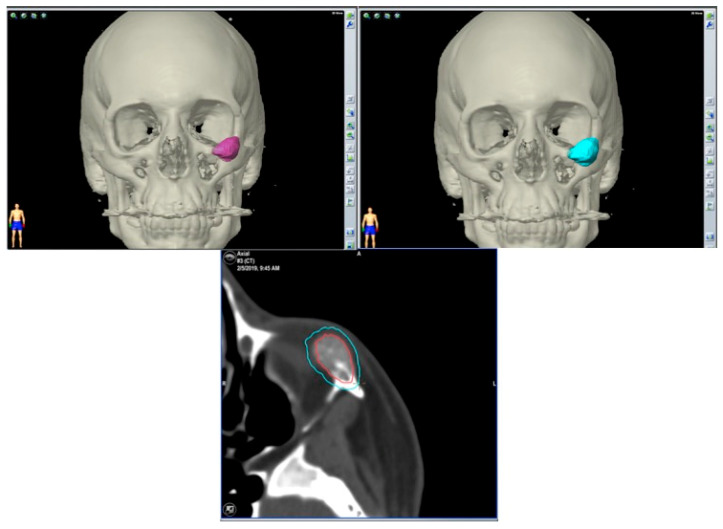
Surgical plan with the BrainLab^®^ navigation software, iPlan® 3.0.6 (Munich, Germany). The lesion is colored in red and the surgical margin in blue.

**Figure 13:**
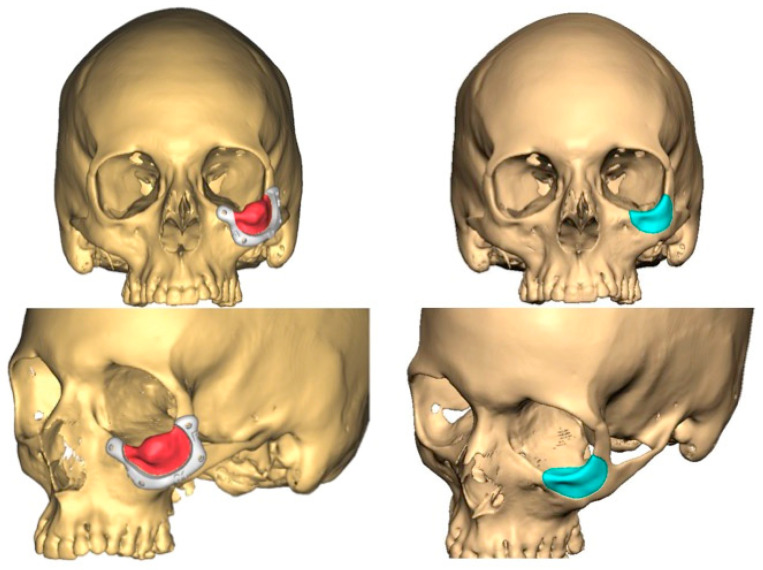
This image shows the lesion (in red), the surgical guide (in white) and the planned PEEK prosthesis (in blue).

**Figure 14:**
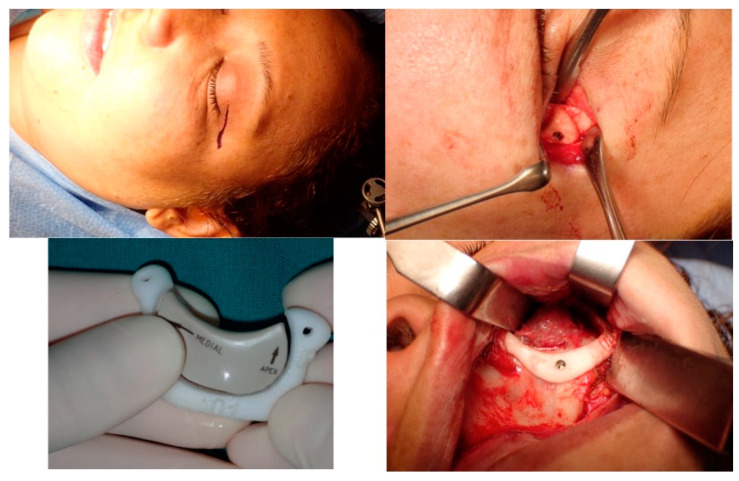
This picture shows the surgical approach to the orbit, the surgical guide and the prosthesis, the vestibular intraoral approach with the surgical guide in position anchored to the zygoma with screws and the superior osteotomy line.

**Figure 15:**
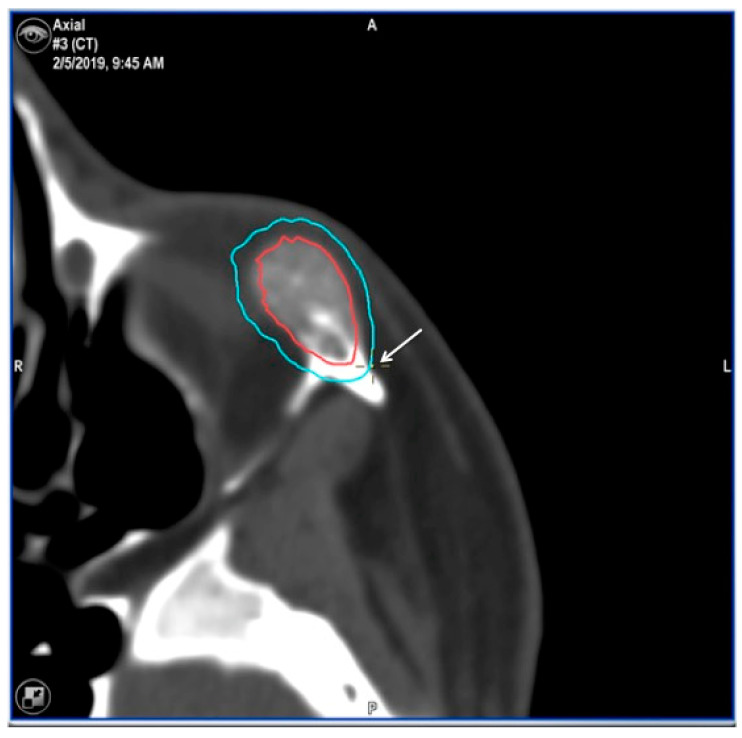
Images of direct navigation with the piezoelectric device on the less-visible posterior part the zygoma (yellow dot with small cross) are shown on the navigation screen. Again, the progression and depth of the guided osteotomy can be appreciated and evaluated in real time (“live navigation”). The lesion is colored in red and the surgical margin in blue.

**Figure 16:**
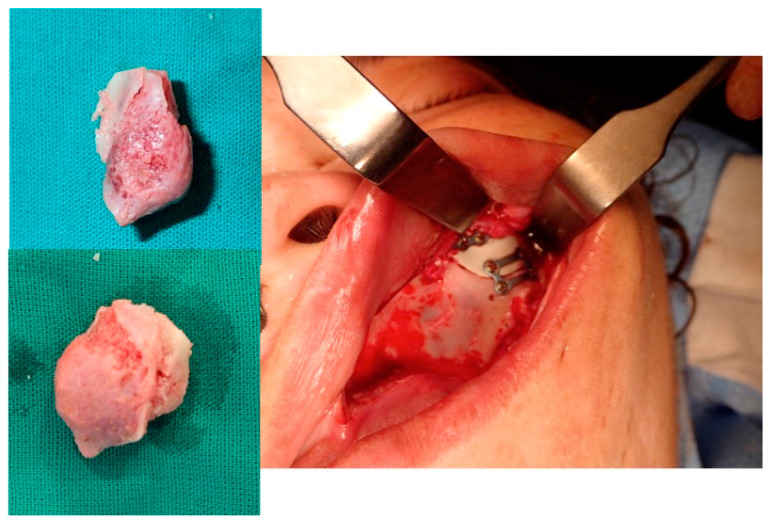
The resected lesion, the PEEK prosthesis and the osteosynthesis with miniplates (intraoral approach).

**Figure 17:**
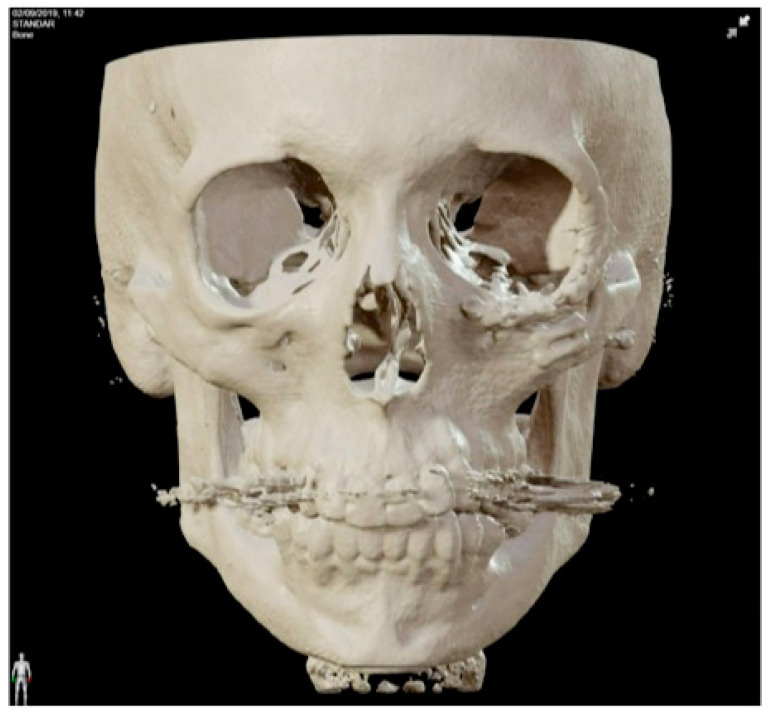
Postoperative 3D CT.

**Figure 18:**
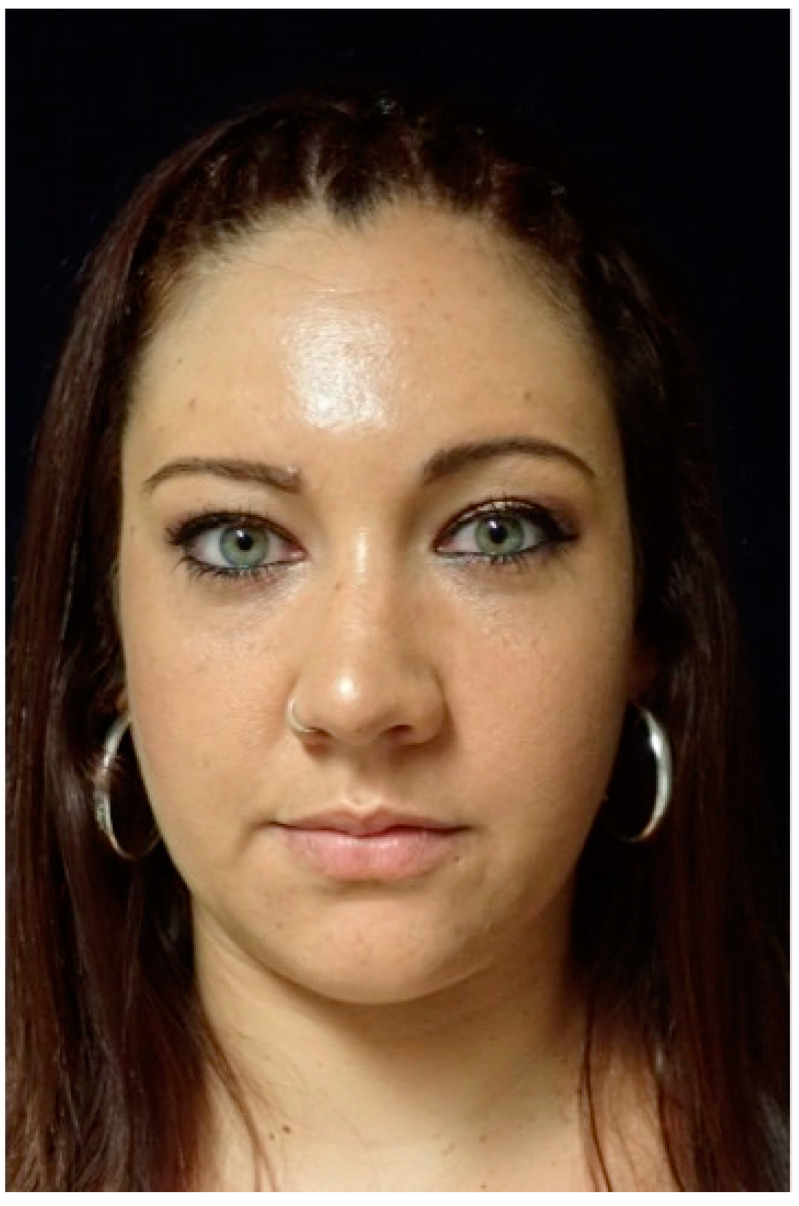
Postoperative facial appearance.

**Table 1:** Patient data, clinical symptoms, locations, histopathology, virtual surgery, surgical navigation, reconstruction.

Case	Age/Sex	Side/Size	Pain	Time of Evolution	Ocular Symptoms	History of Trauma	Imaging	Localization	Histopathology	GLUT-1	Treatment	Virtual Planning	Surgical Navigation	Type of Surgical Navigation	Surgical Approach	Surgical Guides	Surgical Device (Bone Resection)	Bleeding	Reconstruction	Follow-Up/Recurrence
1	53/F	L/25 mm	Y	4 mo	Dystopia	N	CT, MRI	Supraorbital rim, orbital roof	Intraosseous venous malformation	-	Resection + reconstruction	Y	Y	1st, 2nd, 3rd	Coronal	Y	Piezoelectric device	N	PEEK prosthesis	7 y/N
2	54/F	R/33 mm	Y	9 y	N	Y	CT, MRI	Frontal bone, orbital roof	Intraosseous venous malformation	-	Resection + reconstruction	Y	Y	1st, 2nd, 3rd	Coronal	Y	Piezoelectric device	N	PEEK prosthesis	6 y/N
3	36/F	L/19 mm	Y	6 mo	N	N	CT, MRI	Zygoma	Arteriovenous malformation	-	Resection + reconstruction	Y	Y	1st, 2nd, 3rd	Transconjunctival + blepharoplasty + maxillary vestibular	Y	Piezoelectric device	N	PEEK prosthesis	5 y/N
4	47/M	L/30 mm	N	2 y	N	N	CT	Zygoma	Intraosseous venous malformation	-	Resection + reconstruction	Y	Y	1st, 2nd, 3rd	Transconjunctival + lateral canthotomy + maxillary vestibular	Y	Piezoelectric device	N	PEEK prosthesis	6 y/N

Abbreviations: F, female; M, male; CT, computed tomography; MRI, magnetic resonance imaging; mo, month; y, year; Y, yes; N, no; 1st, first navigation; 2nd, second navigation; 3rd, third navigation.

## Data Availability

The data presented in this study are available on request from the corresponding authors.
